# Comment on 'A conserved strategy for inducing appendage regeneration in moon jellyfish, *Drosophila*, and mice'

**DOI:** 10.7554/eLife.84435

**Published:** 2023-06-22

**Authors:** Anne Sustar, John C Tuthill

**Affiliations:** 1 Department of Physiology and Biophysics, University of Washington Seattle United States; CNRS - Université Paris Cité France; University of Michigan United States

**Keywords:** regeneration, limb, fruit fly, *D. melanogaster*

## Abstract

Abrams et al. report that a simple dietary supplement is sufficient to induce appendage regeneration in jellyfish, fruit flies, and mice (Abrams et al., 2021). This conclusion is surprising because it was previously thought that flies and mice lack the capacity for regeneration after injury. We replicated the *Drosophila* experiments of Abrams et al. but did not observe any instances of leg regeneration. We also conclude that the "white blob" observed at the amputation site by Abrams et al. consists of bacteria and is not regenerated tissue.

## Introduction

Abrams et al., 2021 reported that supplementing an animal’s diet with L-leucine and insulin/sucrose promotes appendage regeneration, even in species that were previously thought to lack the capacity for regeneration. The potential discovery of a universal means to unlock regenerative capacity is exciting because it could be applied to other animals, including humans. Indeed, a central conclusion of Abrams et al. is that their “study suggests that an inherent ability for appendage regeneration is retained in non-regenerating animals and can be unlocked with a conserved strategy.”

We initially became interested in the specific part of the Abrams et al. study that addressed limb regeneration in the fruit fly, *Drosophila melanogaster*. Because we study proprioception and motor control of the *Drosophila* leg, we were curious to investigate how neurons and muscles regenerate in injured limbs. We initially focused on replicating the finding that fruit flies can, rarely and under specific experimental conditions, regenerate amputated legs.

Appendage regeneration has been extensively studied in insects. Some groups of insects have been shown to regenerate whole limbs, while others not at all. In general, regeneration capacity is linked to how an insect develops through metamorphosis. Specifically, molting is a key prerequisite for regeneration (Yang et al., 2016). Hemimetabolous insects, such as stick insects, cockroaches, and crickets, have incomplete metamorphosis – they develop as nymphs that resemble small adults. Nymphs possess miniature versions of adult appendages, including legs, antennae, and in some cases, wings. As the nymph progresses through instars, developmental programs induce the animal to molt, shedding and regrowing its exoskeleton. When the limb of a hemimetabolous insect is amputated, it can presumably reactivate similar developmental patterning pathways and replace missing structures, including ectodermal tissue (Bando et al., 2018; Bodenstein, 1955; Bohn, 1971; French et al., 1976).

In contrast, holometabolous insects, such as flies and butterflies, undergo complete metamorphosis to become adults. They initially develop into larvae in which the adult limb primordia are set aside as imaginal tissues. Cells that will become the adult legs, for example, are specified as distinct progenitor populations during embryogenesis. Later, during larval and/or pupal life, these cells undergo extensive proliferation, and ultimately differentiate and undergo morphogenesis to form the adult ectodermal structures during pupation. During this early developmental phase, prior to differentiation, imaginal discs possess the capacity to regenerate following experimental removal of pieces of tissue (Fox et al., 2020; Haynie and Bryant, 1976; Schubiger, 1971). After the final metamorphosis to adulthood, however, holometabolous insects do not molt, and have never been found to regenerate lost or damaged appendages. The limbs of adult holometabolous insects, such as fly legs, are thought to lack the developmental programs required to re-establish patterning and tissue growth after injury, likely due to epigenetic silencing of developmental genes (Fox et al., 2020; Harris et al., 2020; Repiso et al., 2011).

We began by repeating the fly leg amputation experiments of Abrams et al. We copied the protocol described in their paper and through consultation with the senior author. We used the same wild-type *Drosophila* strain (Canton-S), experimental timeline, amputation site, and dietary conditions. Because Abrams et al. observed regeneration in only ~1% of flies fed the supplemented diet, we used a sample size of ~1,000 flies, comparable to that described in their paper. When we failed to find evidence for cuticle regeneration, we next searched for specific tissues within the amputated leg stump to test whether flies are capable of regenerating neurons, muscle, or any other cell class. We also carefully examined the white blob at the cut site, a structure that Abrams et al. interpreted as an intermediate regeneration morphology.

Overall, we found no evidence for regeneration of any cells in the amputated leg. Rather, we find that upon amputation, the cells in the amputated leg segment all died and did not grow back. Furthermore, we provide evidence that the white blob growing on the amputation site is not a regeneration blastema, but rather a colony of bacteria. Our conclusions are consistent with the past literature supporting a lack of limb regeneration in *Drosophila* and other adult holometabolous insects.

## Results

### Absence of evidence for regeneration after amputation of *Drosophila* legs

**Figure 1.**
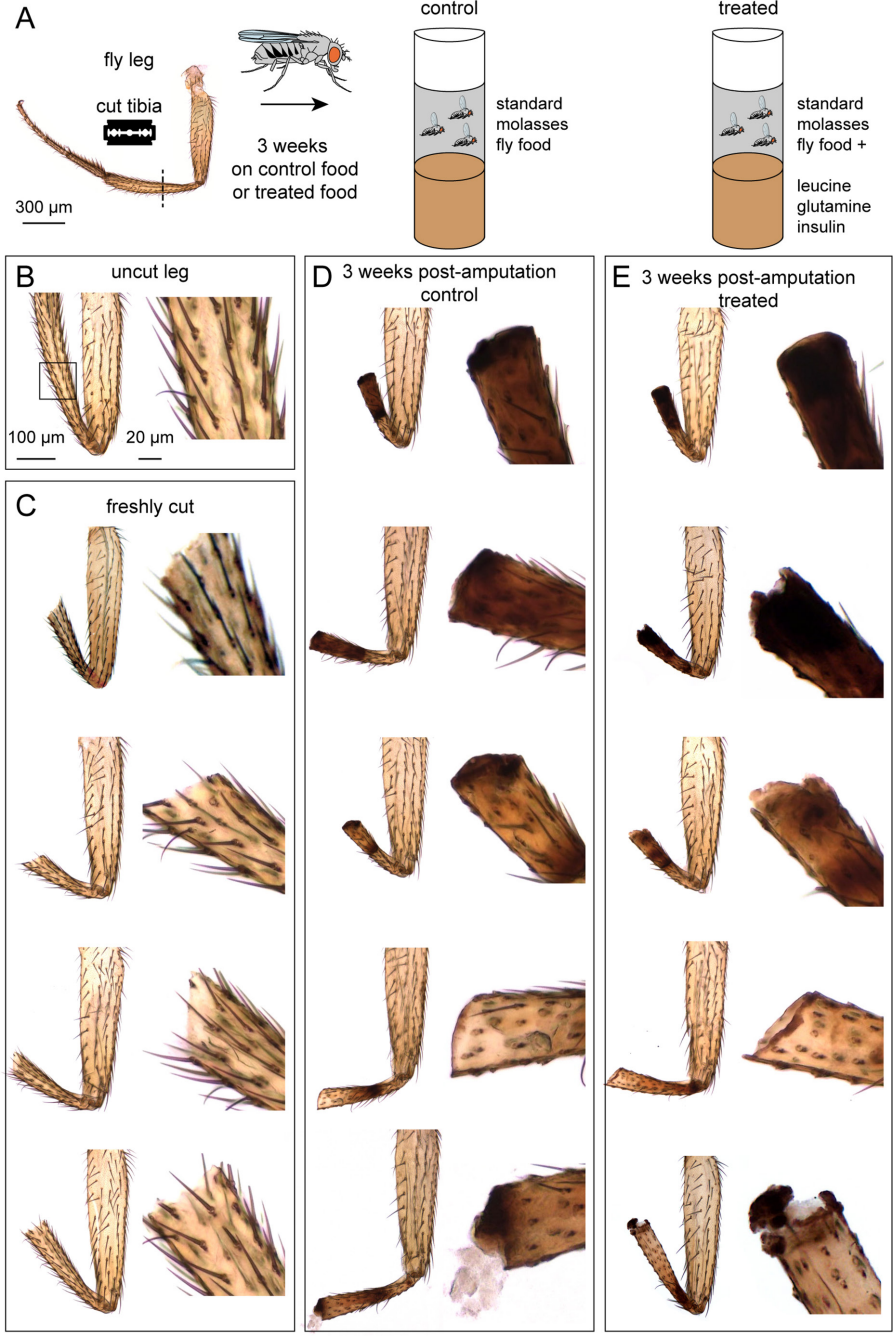
Tibia amputation in wild-type (Canton-S) *Drosophila* legs. (**A**) The experimental protocol. We amputated one hind leg per fly, at the midpoint of the tibia. After three weeks on control food or treated food, legs were fixed and analyzed. (**B–E**) Bright-field images of a control leg (**B**), four examples of freshly cut legs (**C**), and five examples of legs after three weeks on control food (**D**) or treated food (**E**). Insets showed magnified views of the cut site. Scale bar in B is the same for other panels.

Abrams et al. concluded that fly legs, which normally do not regenerate after amputation, show some regeneration ability when the fly’s diet is supplemented with insulin, leucine, and glutamine. We carefully followed their methods to replicate the fly leg regeneration experiments in Figure 3 of their paper. We amputated legs of 1283 flies, one hind leg per fly, at the midpoint of the tibia (Figure 1; Table 1). The majority of these flies, 1083, were of the same wild-type fly strain (Canton-S) used in their study. After amputation, we raised 240 flies on control food and 843 on treated food. Three weeks later, we examined the legs at high magnification using bright-field microscopy, with the experimenter blind to experimental condition.

**Table 1.** Summary of fly tibia amputation results. Table 1—source data 1.Table of and details of amputation Experiments 1-5.

			Control	n	Treated	n
Wild type (Canton-S)	flies amputated			240		843
	survival after 3 weeks			117		498
	tibia stump	cuticle growth	**0%**	0/117	**0%**	0/498
		white blob	**4%**	5/117	**3%**	16/498
		phalloidin stain	**0%**	0/97	**0%**	0/452
		EdU stain	**0%**	0/20	**0%**	0/46
sensory neuron GFP reporter (ChAT >GFP)	flies amputated			100		100
	survival after 3 weeks			64		50
	tibia stump	cuticle growth	**0%**	0/64	**0%**	0/50
		white blob	**0%**	0/64	**4%**	2/50
		GFP	**0%**	0/64	**0%**	0/50

We did not observe any regrown tibias, either in the control group or the treated group. The outcome of the two groups was qualitatively similar (Figure 1). All tibia stumps had bristle deterioration and darkened cuticle, indicating necrosis. The site of the darkened cuticle varied. It was usually near the cut site, but sometimes farther up the leg, closer to the tibia-femur joint. In 4% of control cases and 3% of treated cases, we observed a white blob near the cut site (Table 1; Figure 1, **bottom row**). The white blob was also observed by Abrams et al. to occur at a similar frequency and was called “white tissues protruding from the end”. Overall, based on close inspection of the cuticle three weeks after amputation, we found no evidence of leg regeneration.

### All cells die and fail to regenerate in the amputated tibia stump

Most of the internal structures of the fly leg, including muscle and neurons, are partially transparent. Inspection of amputated limbs using bright-field microscopy alone might be insufficient to detect surviving or regenerated tissue. We therefore used fluorescent labels to test for the presence of muscles, neurons, and other cells in the tibia before and after amputation.

Each fly leg has ~500 tactile bristles, including ~120 distributed uniformly along the tibia (Schubiger and Hadorn, 1968). Each bristle is innervated by a single sensory neuron. To ask whether bristle sensory neurons can regenerate after injury, we amputated 200 legs in a fly strain with a fluorescent reporter that labels bristles and other sensory neurons (ChAT-Gal4 >UAS GFP). We raised half of the flies on control food and half on food with the supplemented diet. We then used confocal imaging to image GFP expression in the leg.

**Figure 2.**
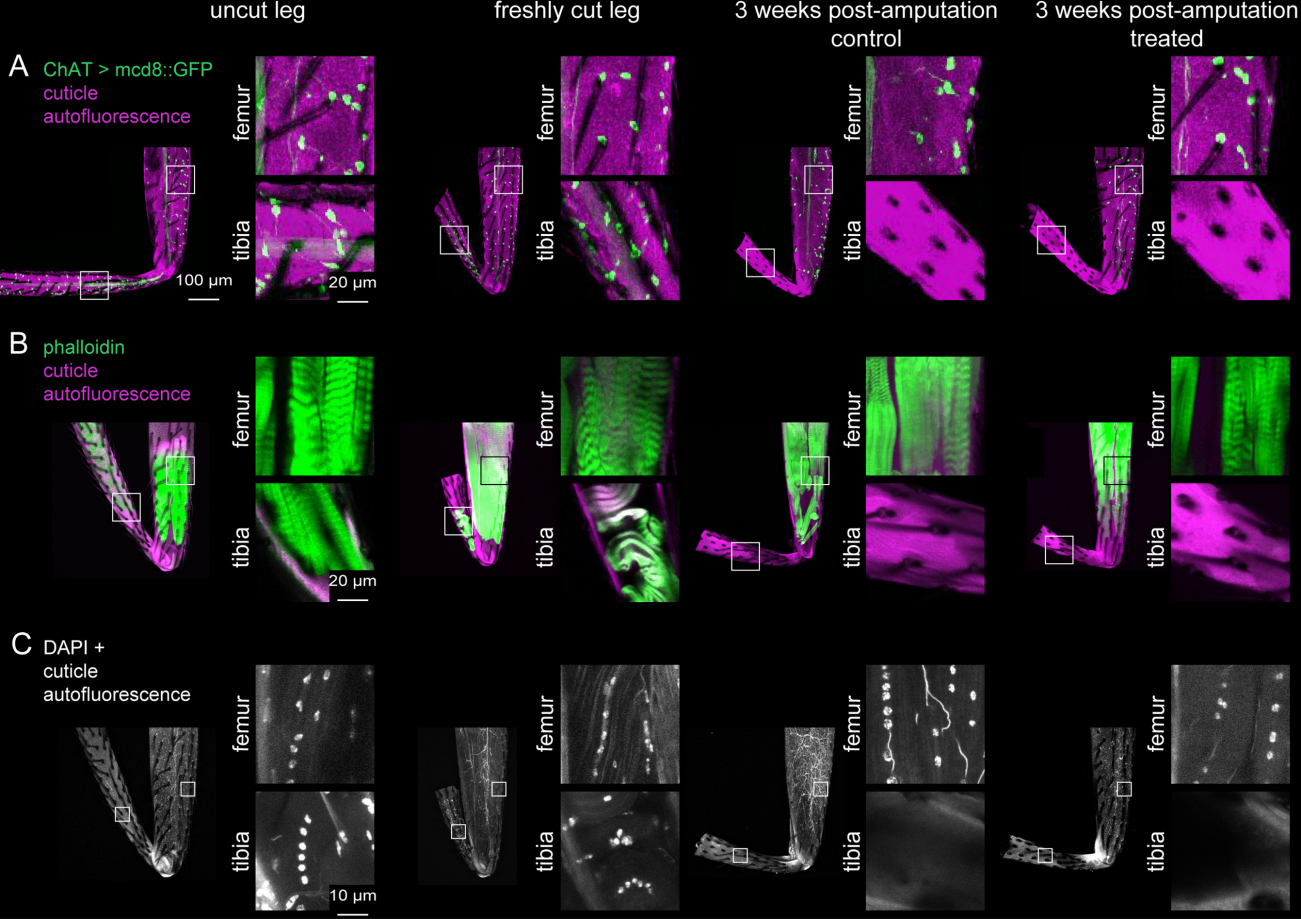
Investigation of tissue identity in amputated legs. Sensory neurons were labeled with ChAT-Gal4 >UAS GFP (**A**), muscles were labeled with phalloidin (**B**), and nuclei were labeled with DAPI (**C**) in uncut legs, freshly cut legs, and 3 weeks post-amputation with control food or treated food. Insets show magnified views of tissue within the femur and tibia. Scale bars in A are the same for all other panels except for insets in C.

Although bristle neurons were present immediately after amputation, they were absent three weeks later, presumably due to deterioration following cell death. The external bristle hairs also deteriorated and did not reappear. The results were indistinguishable between the control and treated groups (Figure 2A; Table 1). In the femur and other leg segments proximal to the tibia, the bristle sensory neurons and hairs appeared normal. In summary, we failed to find any evidence that leg sensory neurons regenerate following tibia amputation.

Each fly leg has twelve muscles, including four in the tibia (Soler et al., 2004). We next tested whether these tibia muscles can regenerate after amputation. We fluorescently labeled wild-type (Canton-S) leg muscles with phalloidin, a stain that labels F-actin. Three weeks after tibia amputation, neither the control group nor the treated group had any muscle staining in the tibia stump (Figure 2B; Table 1). In the femur and other leg segments proximal to the amputation site, muscle staining was normal. In summary, we failed to find any evidence that leg muscles regenerate following tibia amputation.

Since we did not find any sensory neurons or muscles in the amputated tibia stumps, we asked whether any other tissues, possibly hemocytes, glia, or epithelial cells, survive and/or regenerate. We stained legs of wild-type (Canton-S) flies with DAPI to label nuclei. Three weeks after amputation, we did not observe any DAPI staining in the cuticle of the tibia stumps, either in the control or treated groups (n=137 control, n=544 treated; Figure 2C). In the femur and other leg segments proximal to the tibia, DAPI staining looked normal. This pattern is consistent with previous work showing that cell death was constrained to the injured leg segment in the adult cockroach (Bodenstein, 1955). In summary, our evidence supports the conclusion that all cells in the tibia stump die after amputation and fail to regenerate. The amputated stump appears to be an empty tube of cuticle, devoid of living cells.

### The white blob on amputated leg stumps is not a regeneration blastema

Abrams et al. reported the occasional appearance of a white blob at the tip of the amputated tibia stump in flies fed the supplemented diet. They called this “white tissue” and interpreted it to be an intermediate regeneration morphology. We observed the white blob form with a similar probability to that reported by Abrams et al. (3–4% of amputations, Table 1); however, we found that the blob occurred in both the control and experimental groups. Nonetheless, we sought to determine the nature of the white blob, and if it was, in fact, a sign of regeneration.

**Figure 3.**
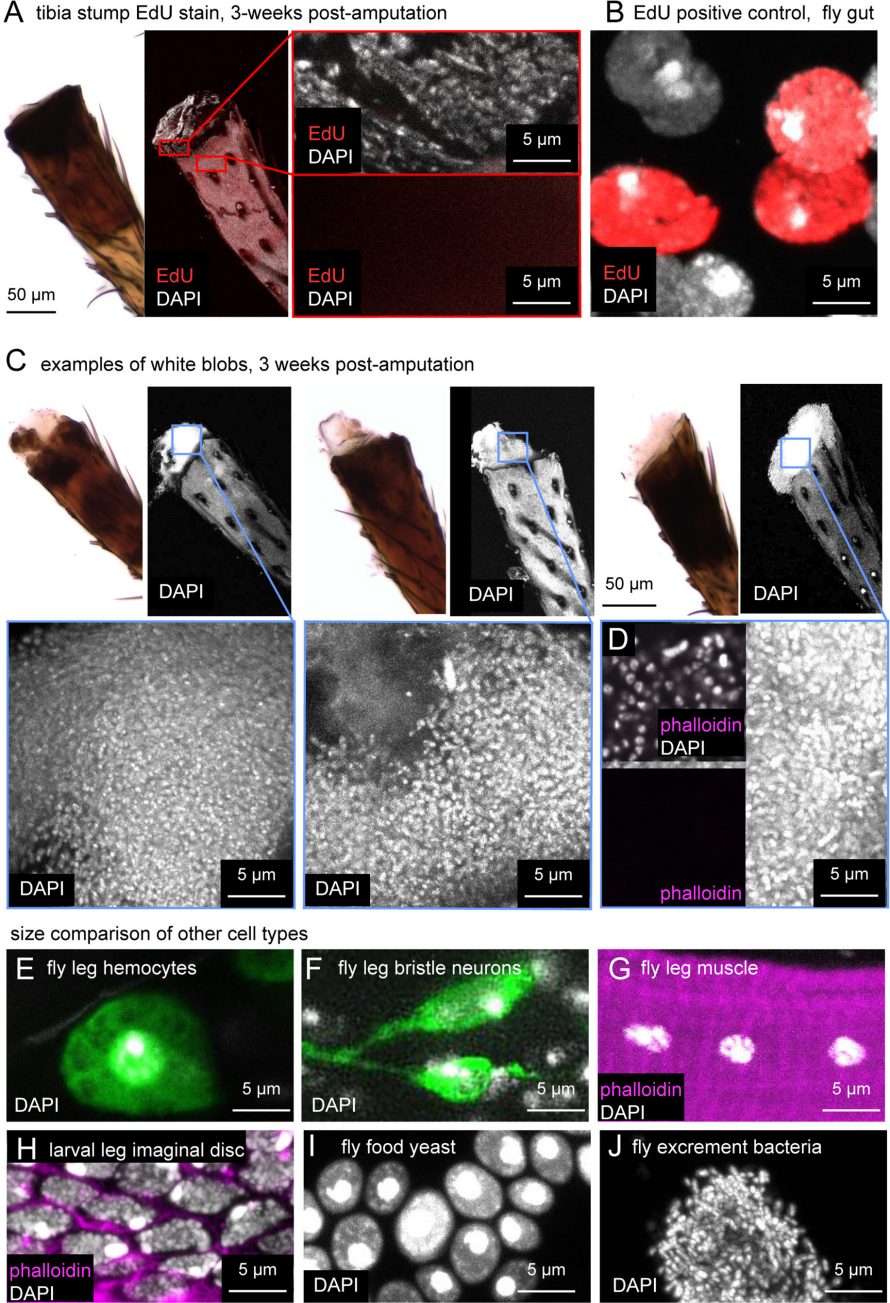
The white blob on amputated tibia stumps is most likely composed of bacteria, not regenerating fly tissue. (**A**) Bright-field (left) and confocal images (right) of leg stumps stained with EdU (red) and DAPI (white) to test for cell proliferation. Tibia stumps did not incorporate EdU. The positive control, fly gut (**B**) did stain for EdU. (**C**) Three additional examples of DAPI staining and one example showing lack of phalloidin staining (**D**) in white blobs (n=12). Note that cells in (**C**) are smaller and more densely packed than fly leg hemocytes (**E**, green = *Hml >* GFP), fly leg bristle sensory neurons (**F**, green = *39A11 Gal4>GFP*), fly leg muscle cells, larval leg imaginal disc cells (**H**), or fly food yeast (**I**). The nuclei in (**C and D**) are consistent with small, densely packed bacteria, such as those observed in fly excrement (**J**).

In many regeneration model systems, tissue regrowth is mediated by a blastema: a concentrated group of undifferentiated cells near the amputation site that proliferates to grow and repattern the missing tissue (Kiehle and Schubiger, 1985; Lehrberg and Gardiner, 2015). One possibility we considered is that the white blob at the cut site in amputated fly legs is a blastema. Alternatively, we thought that the blob could be fly tissue (e.g., muscle) extruded from the leg, a phenomenon which we sometimes observed immediately following amputation. To distinguish between these possibilities, we performed 5-ethynyl-2′-deoxyuridine (EdU) labeling on amputated legs (Figure 3A–B). EdU is a thymidine analog that is incorporated into the DNA of proliferating cells during S-phase. It has been used extensively to label cell proliferation in regeneration blastemas of many animals, including *Drosophila* imaginal discs (Lehrberg and Gardiner, 2015; Worley et al., 2022). We amputated wild-type (Canton-S) fly legs, as above, and fed flies EdU continually for three weeks with either control food or the supplemented diet. When we harvested the legs for EdU staining, we also saved the fly gut as a positive control. Gut is one of the few adult fly tissue types that exhibits homeostatic cell proliferation during the adult stage (Xiang et al., 2017). All fly guts (n=5) had robust EdU staining (Figure 3B). However, none of the tibia stumps had EdU staining (Table 1), including legs with the white blob (Figure 3A). We conclude that the amputated stump lacks regenerating cells and that the white blob is not a regeneration blastema.

### The white blob on amputated leg stumps is likely a colony of bacteria

We found that the white blob stained robustly with the nuclear label DAPI (Figure 3A, C and D). However, the nuclei in the white blobs were about one tenth the size of other cells in the fly leg, including leg hemocytes (Figure 3E), leg sensory neurons (Figure 3F), and leg muscle (Figure 3G). This discrepancy made us doubt that the white blob consisted of *Drosophila* cells. Abrams et al. also performed DAPI staining on this type of structure, but the image in their paper (Figure 4f) lacked a scale bar, making it difficult to determine the source of the nuclei.

In some animals that do regenerate amputated limbs, such as newts and axolotls, blastema cells in an amputated leg de-differentiate, undergoing morphological and transcriptional changes to become more like younger cells (Leigh et al., 2018; Tanaka et al., 2016). To address this possibility in the fly leg, we compared DAPI staining in the white blob to DAPI staining in leg precursor cells of the larval leg imaginal disc (Figure 3H). The nuclei in the white blob were again approximately one tenth the size of those of leg imaginal disc cells. This size difference is inconsistent with the idea that the white blob consists of de-differentiating fly tissue.

We compared DAPI staining of the white blob to other cells present in a *Drosophila* food vial: baker’s yeast and bacteria. The size of baker’s yeast nuclei was comparable to that of fly and other eukaryotic cells — significantly larger than the nuclei in the white blob (Figure 3I). However, the size and density of DAPI labeling of the white blob did resemble DAPI staining of bacteria from fly excrement (Figure 3J).

Finally, to further investigate the possibility that the white blob is a growth of bacteria, we stained the white blob with phalloidin (Figure 3D). If the cells were an outgrowth of fly tissue, we would expect them to have an f-actin-cytoskeleton that stains robustly with phalloidin (Figure 3H). If, on the other hand, the cells are bacteria, we would expect no phalloidin staining because the bacterial cytoskeleton is built from actin homologs, primarily MreB, that do not bind phalloidin (Shih and Rothfield, 2006; Dempwolff et al., 2011). Consistent with the second scenario, we observed that the cells in the white blob did not stain with phalloidin (n=12; Figure 3D). Furthermore, we speculate that the white blob did not incorporate EdU in the earlier experiment (Figure 3A–B) because the bacteria are not feeding on EdU-fly food, but on decomposing fly tissue in the leg stump that is non-proliferating and thus EdU-free. We conclude that the white blob is most likely a colony of bacteria and not an intermediate fly tissue related to regeneration morphology.

## Discussion

Our results support the conclusion that fly legs do not regenerate after amputation. Three weeks after amputation, we found that the tibia is completely devoid of neurons, muscles, and other cell nuclei. The stump thus appears to be simply a tube of hollow cuticle. DAPI and EdU staining of the amputation site confirmed the complete absence of living or regenerating cells in the tibia. Our results were not different between the control group and flies fed supplemental insulin, leucine, and glutamine. Overall, our conclusions are consistent with the prevailing dogma that adult holometabolous insects do not regenerate lost ectodermal structures (Fox et al., 2020; Repiso et al., 2011).

How do we reconcile our results with those of Abrams et al.? We speculate that the conclusions of the original paper were based on inaccurate measurement techniques. Detecting a subtle phenotype that occurs in only 1% of treated flies would require an exceptional degree of measurement accuracy. (Indeed, the sample size in some of our experiments, specifically with ChAT >GFP (Figure 2A, n=114), may also have been too small to capture such rare events.) We propose that Abrams et al. quantification of sub-mm changes in tibia cuticle length lacked the requisite precision. Even so, this explanation fails to account for the differences in limb length distributions when the authors’ tracked limb length over time (e.g., Figure 5f in Abrams et al.). Another contributing factor could be bias in their experimental measurements or analysis. The paper states that, “blind measurements were performed on one pair of control and treated datasets”, but it is not clear how or on what data blinded measurements were performed. Another possibility, which we consider unlikely, is minor differences in experimental methods. For example, we amputated legs with a razor instead of scissors, because we found that using a razor led to a cleaner cut. Although we used the same wild-type strain (Canton-S), different Canton-S sub-strains have been maintained in different laboratories for decades, so one would expect them to have some genetic differences. However, Abrams et al. originally concluded that “Induction of regenerative response was observed across genetic backgrounds, in Oregon R, as discussed, and Canton S wild-type strains”. Indeed, if there is a universal regeneration diet that is conserved from jelly fish to flies to mice, it should also be robust across wild-type sub-strains.

Instead of relying on potentially noisy measurements of limb length over weeks, we used genetic tools and fluorescent labels to search for neurons, muscles, and other regenerated cells in amputated limbs. We found no evidence of living or regenerated cells within the tibia stump. We question how leg cuticle could regrow, even in rare cases, if no detectable living cells remain in the amputated stump. We also question how ingested insulin, one of the key ingredients in the dietary supplement, could affect limb regeneration, because it would be broken down in the gut (Winter, 1923). (Although it is possible it could access the amputated limb directly by physical contact between the stump and the food.) Finally, our evidence supports the conclusion that the blob of white tissue on the distal tip of the amputated tibia, which Abrams et al. claim is an intermediate regeneration morphology, is more likely a growth of bacteria.

Our results cast doubt on the conclusion that a diet supplement induces tibia regeneration in adult *Drosophila*. We did not attempt to reproduce or validate their regeneration results in jellyfish or mice, due to our lack of expertise with these species. Nonetheless, we feel that flaws in the execution and interpretation of the *Drosophila* experiments undermine the conclusion that "an inherent ability for appendage regeneration is retained in non-regenerating animals and can be unlocked with a conserved strategy" (Abrams et al., 2021). Our results, and the past literature on regeneration in insects, do not support this conclusion.

Our motivation to establish the truth about fly leg regeneration is more than academic. Promising experimental results in genetic model organisms, like flies and mice, often motivate experiments in other species, including humans. Because the ingredients in the supplemental diets used by Abrams et al. are already FDA approved, it is conceivable that “regeneration supplements” could be sold without rigorous prior testing. Based on the results in their paper, the authors have applied for a patent on, “Compositions and methods for inducing appendage and limb regeneration”. We feel that their data, and the efficacy of dietary supplements to induce regeneration, require additional scrutiny and independent replication.

## Materials and methods

**Key resources table:** 

Reagent type (species) or resource	Designation	Source or reference	Identifiers	Additional information
Genetic reagent (*D. melanogaster*)	Canton-S wild type	Celeste Berg, UW	N/A	N/A
Genetic reagent (*D. melanogaster*)	Mi{Trojan – Gal4}ChAT[MI04508-TG4.0] CG7715[MI04508-TG4.0-X]	Bloomington 60317	RRID:BDSC_60317; FBti0168134	ChAT-GAL4
Genetic reagent (*D. melanogaster*)	P{pJFRC7-020XUAS-IVS-mCD8::GFP}attP2	Bloomington 32194	RRID:BDSC_32194; FBti0131936	UAS-mcd8::GFP
Genetic reagent (*D. melanogaster*)	P{w[+mC]=Hml-GAL4.Delta}2, P{w[+mC]=UAS-2xEGFP}AH2	Bloomington 30140	RRID:BDSC_30140	Hml >GFP
Genetic reagent (*D. melanogaster*)	P{y[+t7.7] w[+mC]=GMR39 A11-GAL4}attP2	Bloomington 50034	RRID:BDSC_50034	39A11-Gal4
Chemical compound	L-Leucine	Sigma-Aldrich	L8000	5 mM
Chemical compound	L-Glutamine	Sigma-Aldrich	G3126	5 mM
Chemical compound	Insulin (Human Recombinant)	MP Biomedicals	0219390080	0.1 mg/ml
Chemical compound	Alexa Fluor 647 Phalloidin	ThermoFisher Scientific	A22287	1:50 in PBST
Chemical compound	EdU	Abcam	146186	2 mg/mL in food
Commercial assay or kit	Click-&-Go Plus EdU 555 Cell Proliferation Assay Kit	Click Chemistry Tools	1351	N/A
Chemical compound	VECTASHIELD Antifade Mounting Medium	Vector Laboratories	H-1000–10	
Chemical compound	VECTASHIELD Antifade Mounting Medium with DAPI	Vector Laboratories	H-1200–10	
Software, algorithm	FIJI	PMID:22743772	RRID:SCR_002285	

### Table of genotypes

**Table:** 

Figure 1, Figure 2B–D, Figure 3A–C, F–G	Wild type *Drosophila melanogaster* (Canton-S)
Figure 2A	w[*]; Mi{Trojan-GAL4.0}ChAT[MI04508-TG4.0] CG7715[MI04508-TG4.0-X]/ P{pJFRC7-020XUAS-IVS-mCD8::GFP}attP2
Figure 3D	w[1118]; P{w[+mC]=Hml GAL4.Delta}2, P{w[+mC]=UAS-2xEGFP}AH2
Figure 3E	w[1118]; P{pJFRC7-020XUAS-IVS-mCD8::GFP}attP40; P{y[+t7.7] w[+mC]=GMR39 A11-GAL4}attP2

### Amputation and diet

*Drosophila* were raised on a standard cornmeal-molasses-yeast food fly food at 25 °C with a 14 hr dark/10 hr light cycle. We used male and female adults, 1–2 days post-eclosion, reasoning that young flies would be more likely to regenerate and more likely to survive the three-week recovery period than old flies. For leg amputation, flies were anesthetized in groups of 20 on CO_2_ plates for 5 minutes or less. One hind-leg per fly was amputated at the mid-point of the tibia with a fine double-edge super-stainless razor blade (ASR 72–003). Amputated flies were included in our analysis only if the amputation site was within ~50 μm of the tibia midpoint, using leg bristles as fiducial markers. Regeneration of cuticle was assessed according to whether the tibia length three weeks later fell outside of that range. After amputation, flies were immediately returned to a vial with either standard lab food or treated food, with random assignment.

To make treated food, vials of standard fly food were microwaved to liquefy the food. Before adding supplements, we let it cool to lukewarm to prevent the insulin from denaturing (Kaufmann et al., 2021). We added supplements in an aqueous stock solution and mixed the food for a final homogeneous concentration of 5 mM L-Leucine, 5 mM L-Glutamine, and 0.1 mg/ml insulin (Abrams et al., 2021). Food was mixed and allowed to set at room temperature for one hour. Flies were moved onto freshly prepared food every 2–3 days.

### Fixing, staining, and analysis

Legs or imaginal discs were fixed in 4% formaldehyde (PFA) PBS solution for 20 min followed by rinsing in PBS with 0.2% Triton X-100 (PBT) three times. To label muscle, legs were incubated in 1:50 phalloidin in a PBS solution with the following reagents to improve tissue penetrance: 1% triton X-100, 0.5% DMSO, 0.05 mg/ml Escin (Sigma-Aldrich, E1378), and 3% normal goat serum. Legs were allowed to incubate for one week at 4 °C with occasional rocking. After staining, legs were rinsed 3 x with PBS-Tx, 1 x with PBS, and mounted onto slides in Vectashield with or without DAPI.

Each slide was labeled according to experimental condition. Prior to analysis, we taped-over the labels. Categorizations in Table 1 were performed with the experimenter blinded to experimental condition.

### DAPI staining of yeast and fly excrement bacteria

Cells were transferred to a slide, diluted in water, flame-fixed, then mounted in Vectashield with DAPI.

### EdU labeling

We supplemented the food recipe above with 2 mg/mL EdU. Flies were moved to freshly prepared food every 2–3 days. After three weeks, legs and guts (positive control) were dissected and fixed in 4% formaldehyde (PFA) PBS solution for 20 min and processed according to Click-&-Go kit instructions. After staining, legs were rinsed 3 x with PBS-Tx, 1 x with PBS, and mounted in Vectashield with DAPI.

### Imaging

Mounted legs were imaged on a Confocal Olympus FV1000 (phalloidin, ChAT, EdU, and cuticle autofluorescence images) Leica DMI6000 Widefield (brightfield images), and Leica SP8X (DAPI images). Image stacks were processed in FIJI (Schindelin et al., 2012). Bright-field images were processed in Photoshop with the color channel mixer to correct a bluish background to truer white background.

## Data Availability

Table 1—source data 1 contains the numerical data used to generate Table 1.

## Additional files

MDAR checklist

## References

[bib1] Abrams MJ, Tan FH, Li Y, Basinger T, Heithe ML, Sarma A, Lee IT, Condiotte ZJ, Raffiee M, Dabiri JO, Gold DA, Goentoro L (2021). A conserved strategy for inducing appendage regeneration in moon jellyfish, *Drosophila*, and mice. eLife.

[bib2] Bando T, Mito T, Hamada Y, Ishimaru Y, Noji S, Ohuchi H (2018). Molecular mechanisms of limb regeneration: insights from regenerating legs of the cricket *Gryllus bimaculatus*. The International Journal of Developmental Biology.

[bib3] Bodenstein D (1955). Contributions to the problem of regeneration in insects. Journal of Experimental Zoology.

[bib4] Bohn H (1971). [Intercalary regeneration and segmental gradients in the extremities of Leucophaea larvae (Blattaria) : III. The origin of the intercalary regenerate]. Wilhelm Roux Arch. Für Entwicklungsmechanik Org.

[bib5] Dempwolff F, Reimold C, Reth M, Graumann PL (2011). *Bacillus subtilis* MreB orthologs self-organize into filamentous structures underneath the cell membrane in a heterologous cell system. PLOS ONE.

[bib6] Fox DT, Cohen E, Smith-Bolton R (2020). Model systems for regeneration: *Drosophila*. Development.

[bib7] French V, Bryant PJ, Bryant SV (1976). Pattern regulation in epimorphic fields: cells may make use of a polar coordinate system for assessing their positions in developing organs. Science.

[bib8] Harris RE, Stinchfield MJ, Nystrom SL, McKay DJ, Hariharan IK (2020). Damage-responsive, maturity-silenced enhancers regulate multiple genes that direct regeneration in *Drosophila*. eLife.

[bib9] Haynie JL, Bryant PJ (1976). Intercalary regeneration in imaginal wing disk of *Drosophila melanogaster*. Nature.

[bib10] Kaufmann B, Boulle P, Berthou F, Fournier M, Beran D, Ciglenecki I, Townsend M, Schmidt G, Shah M, Cristofani S, Cavailler P, Foti M, Scapozza L (2021). Heat-stability study of various insulin types in tropical temperature conditions: New insights towards improving diabetes care. PLOS ONE.

[bib11] Kiehle CP, Schubiger G (1985). Cell proliferation changes during pattern regulation in imaginal leg discs of *Drosophila melanogaster*. Developmental Biology.

[bib12] Lehrberg J, Gardiner DM (2015). Regulation of axolotl (*Ambystoma mexicanum*) limb blastema cell proliferation by nerves and BMP2 in organotypic slice culture. PLOS ONE.

[bib13] Leigh ND, Dunlap GS, Johnson K, Mariano R, Oshiro R, Wong AY, Bryant DM, Miller BM, Ratner A, Chen A, Ye WW, Haas BJ, Whited JL (2018). Transcriptomic landscape of the blastema niche in regenerating adult axolotl limbs at single-cell resolution. Nature Communications.

[bib14] Repiso A, Bergantiños C, Corominas M, Serras F (2011). Tissue repair and regeneration in *Drosophila* imaginal discs: regeneration in *Drosophila*. Development, Growth & Differentiation.

[bib15] Schindelin J, Arganda-Carreras I, Frise E, Kaynig V, Longair M, Pietzsch T, Preibisch S, Rueden C, Saalfeld S, Schmid B, Tinevez JY, White DJ, Hartenstein V, Eliceiri K, Tomancak P, Cardona A (2012). Fiji: an open-source platform for biological-image analysis. Nature Methods.

[bib16] Schubiger G, Hadorn E (1968). [Auto- and allotypic differentiation in vivo cultivated foreleg blastemas of *Drosophila melanogaster*]. Developmental Biology.

[bib17] Schubiger G (1971). Regeneration, duplication and transdetermination in fragments of the leg disc of *Drosophila melanogaster*. Developmental Biology.

[bib18] Shih Y-L, Rothfield L (2006). The bacterial cytoskeleton. Microbiology and Molecular Biology Reviews.

[bib19] Soler C, Daczewska M, Da Ponte JP, Dastugue B, Jagla K (2004). Coordinated development of muscles and tendons of the *Drosophila* leg. Development.

[bib20] Tanaka HV, Ng NCY, Yang Yu Z, Casco-Robles MM, Maruo F, Tsonis PA, Chiba C (2016). A developmentally regulated switch from stem cells to dedifferentiation for limb muscle regeneration in newts. Nature Communications.

[bib21] Winter LB (1923). On the absorption of insulin from the stomach. The Journal of Physiology.

[bib22] Worley MI, Everetts NJ, Yasutomi R, Chang RJ, Saretha S, Yosef N, Hariharan IK (2022). Ets21C sustains a pro-regenerative transcriptional program in blastema cells of *Drosophila* imaginal discs. Current Biology.

[bib23] Xiang J, Bandura J, Zhang P, Jin Y, Reuter H, Edgar BA (2017). EGFR-dependent TOR-independent endocycles support *Drosophila* gut epithelial regeneration. Nature Communications.

[bib24] Yang Q, Li Z, Li H, Li Y, Yang Y, Zhang Q, Liu X (2016). Comparison of leg regeneration potency between holometabolous *Helicoverpa armigera* (Lepidoptera: Noctuidae) and hemimetabolous *Locusta migratoria manilensis* (Orthoptera: Acrididae). Environmental Entomology.

